# The efficacy and safety of caplacizumab in Japanese patients with immune-mediated thrombotic thrombocytopenic purpura: an open-label phase 2/3 study

**DOI:** 10.1007/s12185-022-03495-6

**Published:** 2022-11-24

**Authors:** Yoshitaka Miyakawa, Kazunori Imada, Satoshi Ichikawa, Hitoji Uchiyama, Yasunori Ueda, Akihito Yonezawa, Shigeki Fujitani, Yoshiyuki Ogawa, Tadashi Matsushita, Hidesaku Asakura, Kenji Nishio, Kodai Suzuki, Yasuhiro Hashimoto, Hidenori Murakami, Sayaka Tahara, Tomoyuki Tanaka, Masanori Matsumoto

**Affiliations:** 1 Department of Hematology, Saitama Medical University Hospital, Saitama, Japan; 2 Department of Hematology, Japanese Red Cross Osaka Hospital, Osaka, Japan; 3 Department of Hematology, Tohoku University Hospital, Sendai, Japan; 4 Department of Hematology, Japanese Red Cross Kyoto Daiichi Hospital, Kyoto, Japan; 5 Department of Hematology/Oncology, Kurashiki Central Hospital, Kurashiki, Japan; 6 Department of Hematology, Kokura Memorial Hospital, Kitakyushu, Japan; 7 Department of Emergency and Critical Care Medicine, St. Marianna University School of Medicine Hospital, Kawasaki, Japan; 8 Department of Hematology, Gunma University Hospital, Maebashi, Japan; 9 Department of Transfusion Medicine, Nagoya University Hospital, Nagoya, Japan; 10 Department of Hematology, Kanazawa University Hospital, Kanazawa, Japan; 11 Department of General Medicine, Nara Medical University Hospital, Kashihara, Japan; 12 Sanofi K.K., Tokyo, Japan; 13 Department of Blood Transfusion Medicine, Nara Medical University, 840 Shijyo-Cho, Kashihara, Nara 634-8522 Japan

**Keywords:** ADAMTS13, Caplacizumab, Single-domain antibody, Thrombotic thrombocytopenic purpura, Von Willebrand factor inhibitor

## Abstract

Caplacizumab is an anti-von Willebrand factor humanized single-variable-domain immunoglobulin fragment whose efficacy and safety in immune-mediated thrombotic thrombocytopenia purpura (iTTP) have been demonstrated in international studies. This prospective, open-label phase 2/3 study evaluated caplacizumab 10 mg administered daily during plasma exchange and for 30 days afterward, in combination with immunosuppressive treatment, in Japanese adults with a clinical diagnosis of iTTP (new or recurrent). The primary endpoint was prevention of iTTP recurrence; key secondary endpoints included time to platelet count response, time to organ damage normalization, and safety. Among 21 treated patients, 1 of 15 (6.7%) evaluable patients developed iTTP recurrence. Median time to normalization was 2.79 days for platelet count and 2.65 days for organ damage markers (*n* = 15). Treatment-emergent adverse events (TEAEs) were mostly mild to moderate in severity; the most frequently reported caplacizumab-related TEAEs were increased alanine aminotransferase, epistaxis, and gastrointestinal hemorrhage (all in 9.5% of patients). At least one bleeding event was reported in 7 of 21 patients (33%). Caplacizumab was effective in Japanese patients with iTTP, with a low rate of iTTP recurrence, rapid normalization of platelet counts and organ damage markers, and no unexpected TEAEs. Trial registration: ClinicalTrials.gov identifier, NCT04074187.

**Supplementary Information:**

The online version contains supplementary material available at 10.1007/s12185-022-03495-6.

## Introduction

Thrombotic thrombocytopenia purpura (TTP) is an acute life-threatening condition characterized by thrombocytopenia, non-immune hemolytic anemia and organ ischemia [1]. TTP develops when there is a severe deficiency of the disintegrin and metalloproteinase with thrombospondin motifs 13 (ADAMTS13) enzyme, which cleaves von Willebrand factor (VWF). Around 5% of cases of TTP are hereditary as a result of a mutation of ADAMTS13, while others are acquired TTP that is mediated through the development of anti-ADAMTS13 autoantibodies [1].

The acquired, or immune-mediated, form of TTP (iTTP) is rare, but more prevalent than the hereditary form [1]. Moreover, iTTP carries a high risk of mortality during the acute episode, and individuals who survive a first iTTP attack are at high risk of further episodes [2]. The risk of relapse is estimated to be about 40–55% over the 7–8 years following an acute event [2, 3], or about 2% per month [4]. In a US registry study, the 20-year mortality rate was approximately 20% among individuals with iTTP [2], and in Japan, the 12-month rate of iTTP-related mortality has been reported as approximately 13% [5].

Approximately 18% of individuals with iTTP are refractory to the standard treatments [6]. As a result, TTP has been designated as an intractable disease by the Japanese Ministry of Health, Labor and Welfare to promote research and to support individuals with iTTP by providing subsidized treatment. In 2020, 361 individuals in Japan received a beneficiary certificate that was issued for the specific treatment of TTP [7]. Until recently, treatment of iTTP was focused on the use of daily therapeutic plasma exchange (TPE) to remove ultra-large VWF and ADAMTS13 inhibitors and provide ADAMTS13, and immunosuppression with corticosteroid and rituximab to suppress anti-ADAMTS13 antibody production [8].

Caplacizumab is a humanized, single-chain dimer, bivalent, variable-domain-only immunoglobulin fragment directed against the A1 domain of VWF, which inhibits the interaction between VWF and glycoprotein Ib-IX-V receptors on platelets [9]. As these antibody fragments are smaller than standard monoclonal antibodies, they are generally more soluble and stable, and show rapid tissue penetration and clearance [10]; this enables them to be administered by subcutaneous injection. The phase 2 TITAN study and phase 3 HERCULES study demonstrated that caplacizumab, in addition to the standard TPE regimen and immunosuppressive treatment, rapidly normalized platelet levels in individuals with iTTP and allowed for earlier discontinuation of TPE compared with placebo [11, 12]. In the phase 3 HERCULES study, the incidence of a composite endpoint of TTP-related death, recurrence of TTP, or a thromboembolic event was lower with caplacizumab than with placebo. However, to date, no prospective studies with caplacizumab have been undertaken in Japanese individuals with iTTP.

Thus, the aim of the current phase 2/3 study was to evaluate the efficacy and safety of caplacizumab, in addition to standard care, in Japanese individuals with iTTP.

## Materials and methods

### Study design

This was a prospective, single-arm, open-label study (NCT04074187) conducted at 11 centers in Japan between October 2019 and May 2021, and sponsored/funded by Sanofi. The primary objective of the study was to evaluate the ability of caplacizumab to prevent the recurrence of iTTP during the overall study period.

The protocol and other relevant documents were approved by an Institutional Review Board prior to study initiation. In addition, the study was conducted in accordance with the protocol and ethical principles outlined in international guidelines including the Declaration of Helsinki, and the International Conference on Harmonization Good Clinical Practice Guidelines, as well as any applicable laws, rules, and regulations. Participants, or their legally authorized representative, provided written informed consent prior to any study procedures being undertaken.

### Participants

The study included male or female Japanese individuals aged ≥ 18 years with a clinical diagnosis of iTTP (initial or recurrent) that required initiation of daily TPE treatment. A confirmed diagnosis of iTTP based on ADAMTS13 activity was not required for study enrollment. The TTP clinical diagnosis was defined as thrombocytopenia (platelet count < 100 × 10^9^/L), microangiopathic hemolytic anemia as evidenced by erythrocyte fragmentation (e.g., presence of schistocytes), and increased levels of lactate dehydrogenase (LDH). Participants could be included if they had received one intense TPE treatment within the preceding 24 h but no more than this. An additional exclusion criterion was a platelet count of ≥ 100 × 10^9^/L, or a serum creatinine level of > 2.3 mg/dL in individuals with a platelet count of > 30 × 10^9^/L (to exclude possible cases of atypical hemolytic uremic syndrome).

### Treatment

Eligible participants received caplacizumab 10 mg once daily, in addition to standard care, throughout the TPE treatment period and for 30 days after completion of TPE. The first caplacizumab dose was administered by intravenous injection at least 15 min prior to TPE, with subsequent doses administered subcutaneously after TPE. After Day 2, caplacizumab was administered subcutaneously after TPE. Standard care was administered according to Japanese guidelines [13] and consisted of TPE (i.e., daily fresh-frozen plasma replacement of 1–1.5 × estimated plasma volume; continued until ≥ 2 days after the platelet count was ≥ 150 × 10^9^/L), corticosteroids (see Supplementary Methods for dosing schedule), and other immunosuppressive treatment, which was allowed for optimizing immunosuppression. After discharge from hospital, participants continued caplacizumab at home after appropriate training.

**Fig. 1:**
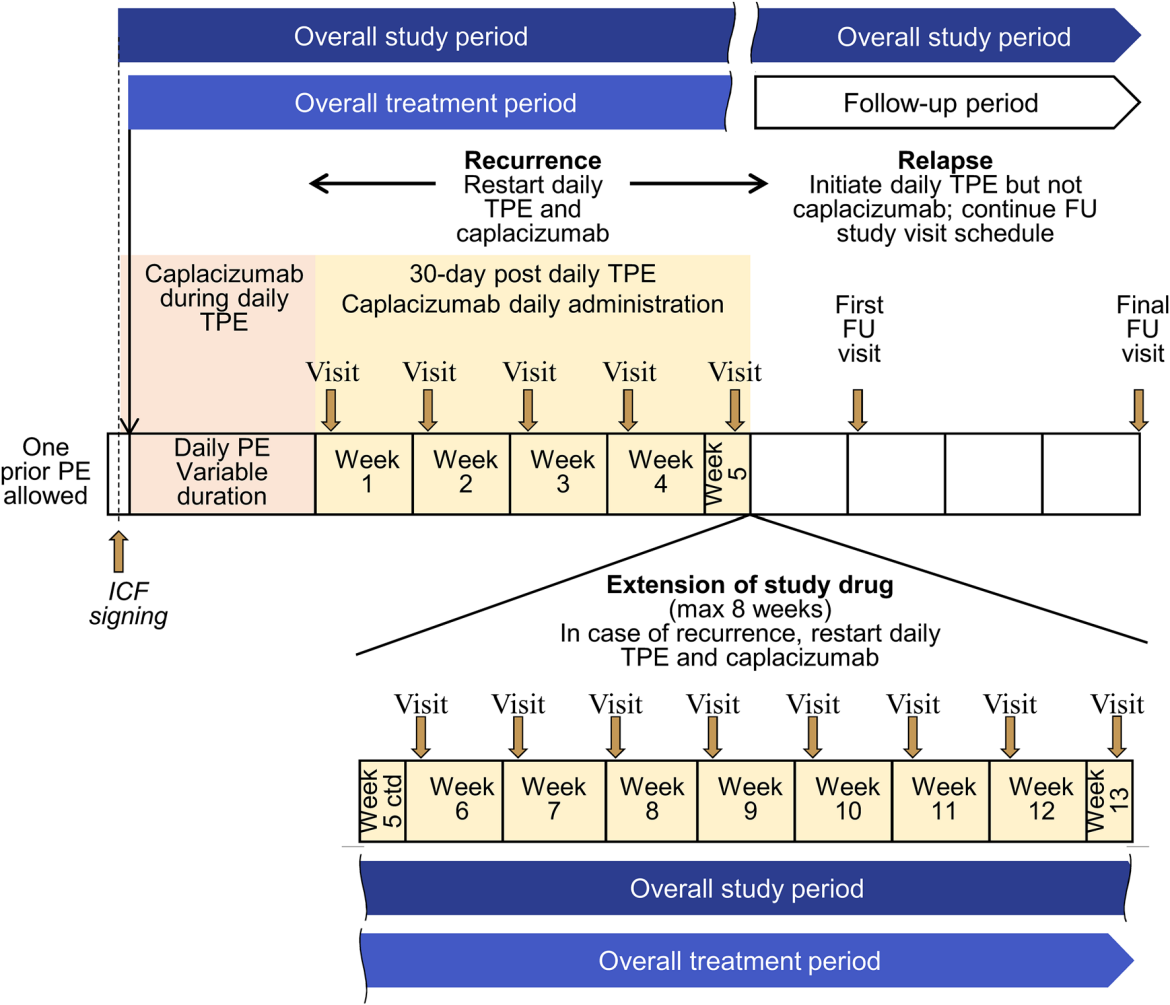
Study design. *Ctd* continued, *FU* follow-up, *ICF* informed consent form, *max* maximum, *PE* plasma exchange, *TPE* therapeutic plasma exchange

The minimum study period was the duration of daily TPE treatment plus 30 days of post-TPE treatment, and 30 days of follow-up period, but this could be extended if participants did not have resolution of the underlying autoimmune disease (Fig. 1). Caplacizumab was continued for additional 1-week periods in participants whose ADAMTS13 activity profile remained < 10% or who had other clinical signs of underlying immunologic disease. The maximum extension period was 8 weeks. Thus, the study period could range from approximately 2–6 months. The decision to reinitiate TPE was based on the participant’s risk of relapse during optimized immunosuppression, assessed using the ADAMTS13 activity profile (measured weekly).

### Endpoints

The primary endpoint of the study was the proportion of participants with a recurrence of iTTP during the overall study period and was assessed in the per-protocol (PP) population. Recurrence was defined as recurrent thrombocytopenia after initial recovery of platelet count (confirmed platelet count ≥ 150 × 10^9^/L with discontinuation of daily TPE), requiring re-initiation of daily TPE. The success criterion was a recurrence rate of ≤ 20%.

Secondary endpoints, assessed in the PP and modified intention-to-treat (mITT) populations, included the composite endpoint of iTTP-related death, iTTP recurrence, or ≥ 1 treatment-emergent major thromboembolic event; the proportion of participants with refractory iTTP (persistent thrombocytopenia, lack of a sustained platelet count increment or platelet count < 50 × 10^9^/L and persistently elevated LDH levels despite five TPEs and corticosteroids); the time to platelet count response (defined as platelet count ≥ 150 × 10^9^/L with subsequent discontinuation of TPE within 5 days); the time to normalization of three organ damage markers (LDH, cardiac troponin l and plasma creatinine). Additional secondary endpoints were the number of TPE days, total volume of plasma (absolute and normalized), and the number of days in ICU and hospital and effects on cognitive function in the PP population; treatment-emergent adverse events (TEAEs, evaluated in the safety population); caplacizumab pharmacokinetics; and the effects of caplacizumab on pharmacodynamic markers [i.e., VWF antigen (VWF:Ag), coagulation factor VIII clotting activity (FVIII:C), and VWF ristocetin cofactor (VWF:RCo)].

### Outcome assessments

The use of TPE and other medications, as well as bleeding events, platelet count, and organ damage markers, were recorded daily until the end of TPE, weekly until the end of the study, and at the first and last follow-up visits. In addition, the Standardized Mini-Mental State Examination (SMMSE) [14] was used to measure cognitive function. SMMSE is a brief 30-point questionnaire, in which total scores of 26–30 are considered normal, 20–25 indicates mild cognitive impairment, 10–20 indicates moderate cognitive impairment, and 0–9 indicates severe cognitive impairment; an SMMSE score of 0 indicates unconsciousness. SMMSE was assessed on Day 1, then during Weeks 1 and 5, and at the first and last follow-up visits. The SMMSE could also be conducted on Days 2, 3 and 4, but this was optional. TEAEs were classified according to the Medical Dictionary for Regulatory Activities (MedDRA) system organ class (SOC) affected and preferred term (PT). The severity of TEAEs (i.e., “mild”, “moderate”, or “severe”) was determined as described in the Supplementary Methods.

With regards to caplacizumab pharmacokinetics, total caplacizumab plasma concentrations were evaluated on Days 2 and 3, then weekly thereafter. Pharmacodynamic parameters (i.e., VWF:Ag, FVIII:C, and VWF:RCo) were evaluated on Days 1, 2, and 3 of the daily TPE period, and then daily during the rest of the follow-up up period. ADAMTS13 activity was evaluated on Day 1 of the daily TPE period, then weekly until the end of the study, and at the first and last follow-up visits. A description of other assessments is provided in the Supplementary Methods.

### Statistical analysis

The planned sample size was 15 participants, based on feasibility considerations; no sample size calculation was performed. The success criterion for the study was ≤ 20% of evaluable participants (i.e., PP population) with a recurrence of iTTP during the overall study period. The PP population (the primary analysis population) included all participants in the mITT who completed treatment and follow-up, or had an iTTP recurrence, and no major protocol deviations. Any individual who was found to have a disease other than iTTP after enrollment was excluded from the PP population. The mITT and safety populations were both defined as all participants who received at least one dose of caplacizumab.

In general, all endpoints were summarized descriptively using total numbers, means, standard errors (SE), medians, confidence intervals (CIs), and ranges (minimum–maximum) for continuous variables, and frequencies and percentages for categorical variables. Time-to-event data were summarized using Kaplan–Meier estimates.

## Results

### Participants

Twenty-one participants were enrolled and received at least one dose of caplacizumab (mITT and safety populations). Six participants discontinued caplacizumab treatment, either because of physician decision (*n* = 4) or TEAEs (pulmonary alveolar hemorrhage and abnormal hepatic function, *n* = 1 each). The PP population, therefore, comprised 15 participants. Baseline demographics and clinical characteristics were generally similar in the mITT and PP populations (Table 1); all participants in the PP population had ADAMTS13 activity < 10% at baseline. The mITT population included 11 men and 10 women; participants were aged 22–86 years (median 59 years). In the mITT population, 16 participants (76.2%) presented with an initial iTTP episode and median (range) platelet count at baseline of 21.5 (8–78) × 10^9^/L (Table 1).

**Table 1:** Baseline demographics and clinical characteristics

	mITT population (*N*=21)	PP population (*N*=15)
Sex, *n* (%)		
Male	11 (52.4)	6 (40.0)
Female	10 (47.6)	9 (60.0)
Age, years, median (range)	59.0 (22–86)	52.0 (22–84)
Weight, kg, median (range)	62.9 (37.9–103.5)	65.8 (43.6–103.5)
BMI, kg/m^2^, median (range)	24.7 (17.3–36.2)	24.7 (18.4–36.2)
Time since TTP diagnosis, days, median (range)	2.0 (1–299)	1 (1–299)
Initial TTP episode, *n* (%)	16 (76.2)	12 (80.0)
Number of previous TTP episodes, *n* (%)		
0	16 (76.2)	12 (80.0)
1	5 (23.8)	3 (20.0)
Platelet count, x10^9^/L, median (range)	21.5 (8–78)	23.0 (8–70)
ADAMTS13 activity level, *n* (%)		
<10%	18 (85.7)	15 (100.0)
≥10%	3 (14.3)	0
LDH, U/L median (range)	581 (227–1929)	575 (227–1794)
Cardiac troponin I level, μg/L, median (range)	0.12 (0.03–7.47)	0.12 (0.03–3.52)
Serum creatinine level, μmol/L, median (range)	79.5 (49.0–294.0)	67.0 (49.0–226.0)
GCS score, *n* (%)		
≤12	3 (14.3)	2 (13.3)
13–15	18 (85.7)	13 (86.7)
SMMSE score, mean (range)	17.3 (0–30)	17.7 (0–30)
Severity of disease, *n* (%)		
Very severe^a^	13 (61.9)	9 (60.0)
Less severe	8 (38.1)	6 (40.0)

*ADAMTS13* a disintegrin and metalloproteinase with a thrombospondin type 1 motif, member 13; *BMI* body mass index, *GCS* Glasgow coma scale, *LDH* lactate dehydrogenase, *mITT* modified intention-to-treat, *PP* per protocol, *SMMSE *standardized Mini-Mental State Examination; *TTP* thrombotic thrombocytopenia purpura

^a^French severity score ≥3, severe neurologic involvement (e.g., coma, seizures, focal deficit), or cardiac involvement (cardiac troponin I level > 2.5 x upper limit of normal).

Among the mITT population, the median duration (range) of caplacizumab exposure during the overall treatment period was 35 (7–69) days. The median compliance rate was 97.1%, with 100.0% compliance in the daily TPE period and 96.7% compliance in the post-daily TPE period. Caplacizumab treatment was extended beyond the planned treatment period by ≥ 1 week in seven participants (≤ 4 weeks in six participants and 5 weeks in one participant). All mITT participants received concomitant corticosteroids, and 10 (47.6%) received rituximab.

### Efficacy

#### iTTP outcomes

**Fig. 2:**
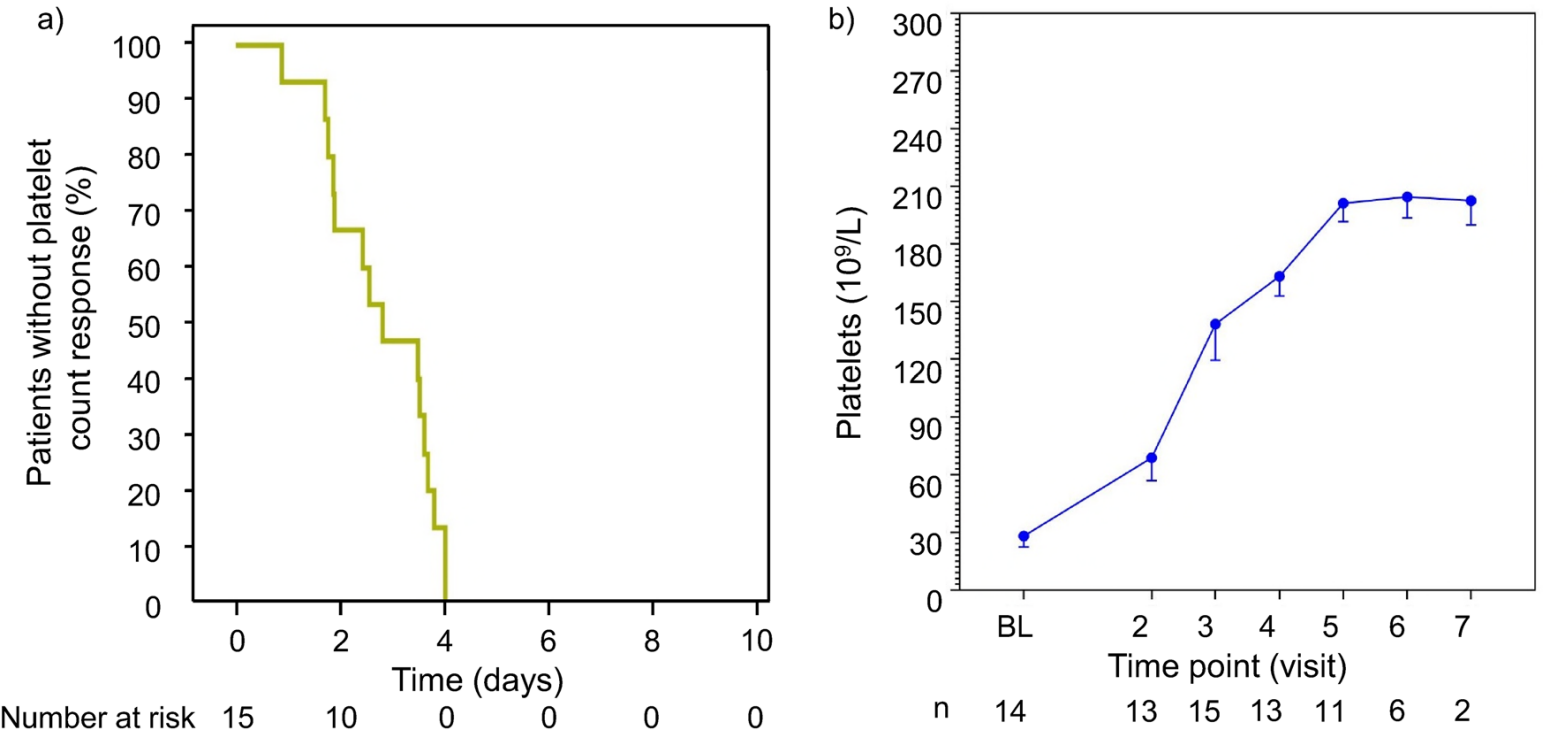
**a** Kaplan–Meier plot of time to platelet count response (per-protocol population) and **b** mean platelet count during daily therapeutic plasma exchange (TPE) period (per-protocol population). In **b**, error bars represent the standard error of the mean. *BL* baseline

One participant developed an iTTP recurrence during the study period, representing 6.7% of the PP population (Table 2), which met the success criterion of ≤ 20%. The recurrence occurred 2 days after caplacizumab had been interrupted (7 days after caplacizumab initiation); caplacizumab had been interrupted, because the participant developed a serious TEAE (i.e., abnormal hepatic function that was considered unrelated to treatment). This participant received a second treatment after the serious TEAE resolved and completed the study. No participants had refractory disease or a major thromboembolic event, and no participants died from TTP during the overall study period in the PP population. In the PP population, the median time to platelet count response was 2.79 (95% CI 1.76–3.59) days (Fig. 2a), with the platelet count increasing rapidly after initiating treatment (Fig. 2b). Major thromboembolic events occurred in two participants [9.5%; deep vein thrombosis (*n* = 1) and cerebrovascular accident (*n* = 1)] in the mITT population. Seventeen participants (81.0%) in the mITT population had a platelet count response; the median time to response in this group was 3.49 (95% CI 2.42–3.98) days.

**Table 2:** Efficacy outcomes

	mITT population (*N*=21)	PP population (*N*=15)
Primary endpoint: recurrence of iTTP,^a^*n* (%)	1 (4.8)	1 (6.7)
Composite endpoint,^b^*n* (%)	3 (14.3)	1 (6.7)
TTP-related death	0	0
iTTP recurrence	1 (4.8)	1 (6.7)
≥1 treatment-emergent TE	2 (9.5)	0
Refractory disease,^c^*n*	0	0
Time to platelet count response, ^d^days		
Median (95% CI)	3.49 (2.42–3.98)	2.79 (1.76–3.59)
25% quantile (95% CI)	2.42 (0.88–2.79)	1.85 (0.88–2.54)
75% quantile (95% CI)	3.98 (3.59–NC)	3.65 (2.79–3.98)
Median time to normalization of organ damage markers (95% CI), days		
All 3 markers	3.57 (1.77–10.78)	2.65 (0.98–4.98)
LDH	–^h^	1.88 (0.87–4.48)^e^
cTnl	–^h^	2.26 (1.48–11.40)^f^
Serum creatinine	–^h^	1.88 (0.49–4.40)^g^
Median number of days of TPE (range),^b^days	–^h^	5.0 (3–7)
Median estimated plasma volume (range),^b^L	–^h^	24.6 (13.4–50.0)

*CI* confidence interval, *cTnI* cardiac troponin I, *iTTP *immune-mediated thrombotic thrombocytopenic purpura, *LDH* lactate dehydrogenase, *mITT* modified intention-to-treat, *NC* not calculated, *PP* per-protocol, *TE* thromboembolic event, *TPE* therapeutic plasma exchange

^a^During overall study period (treatment period + follow-up period)

^b^During overall treatment period

^c^During the 5 days from the first TPE

^d^Platelet count ≥150 ×10^9^/L with subsequent stop of daily TPE within 5 days

^e^*n*=13

^f^*n*=8

^g^*n*=6

^h^No data for mITT population

#### Organ function parameters and cognitive function

**Fig. 3:**
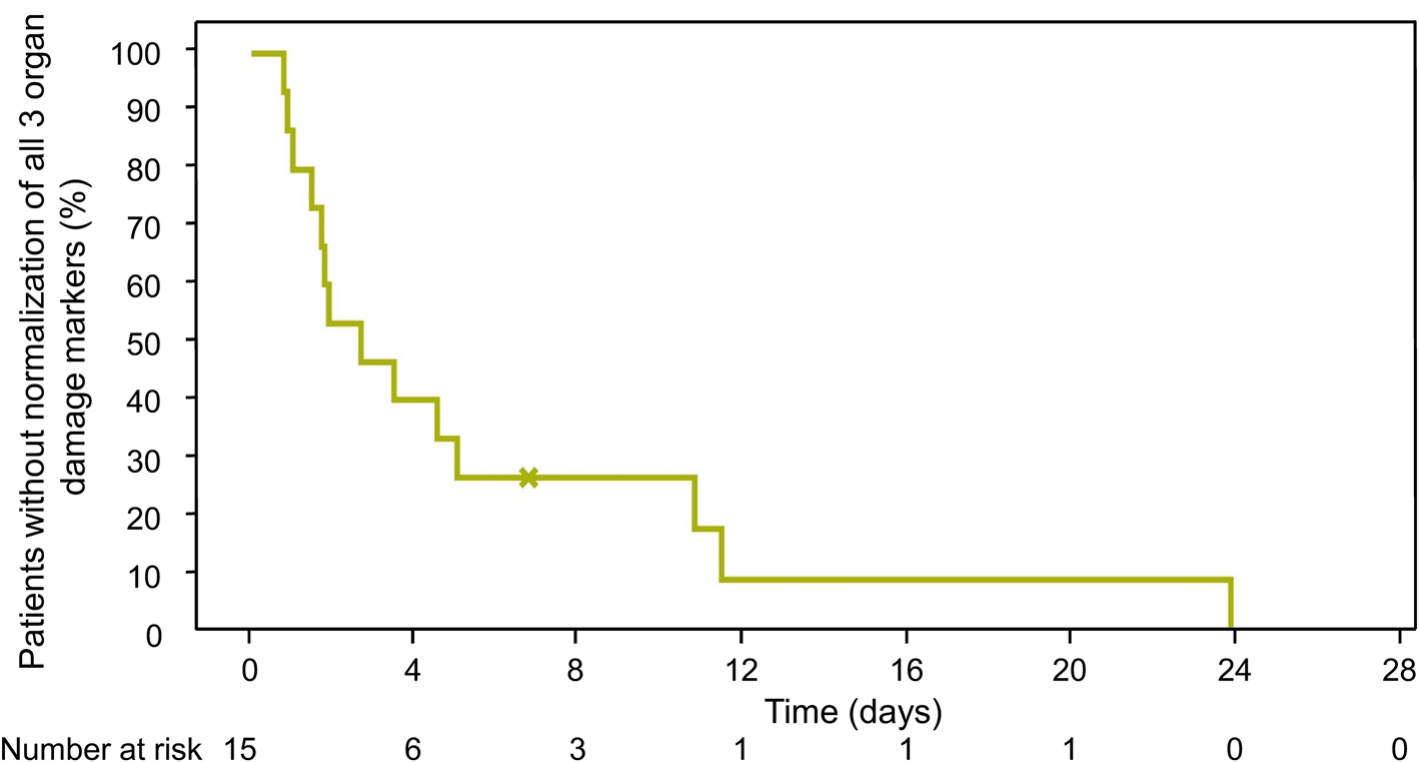
Kaplan–Meier plot of time to normalization of all three markers of organ damage (lactate dehydrogenase, cardiac troponin I, and serum creatinine) in the per-protocol population. The x indicates a censored event

In the PP population, 14 of 15 (93.3%) participants experienced normalization of all three organ damage maker levels, with the median time to normalization in these individuals occurring at 2.65 (95% CI: 0.98–4.98) days (Fig. 3). In the mITT population, normalization of the levels of all three organ damage makers was observed for 18 of 21 participants, and the median time to normalization was 3.57 (95% CI: 1.77–10.78) days.

**Fig. 4:**
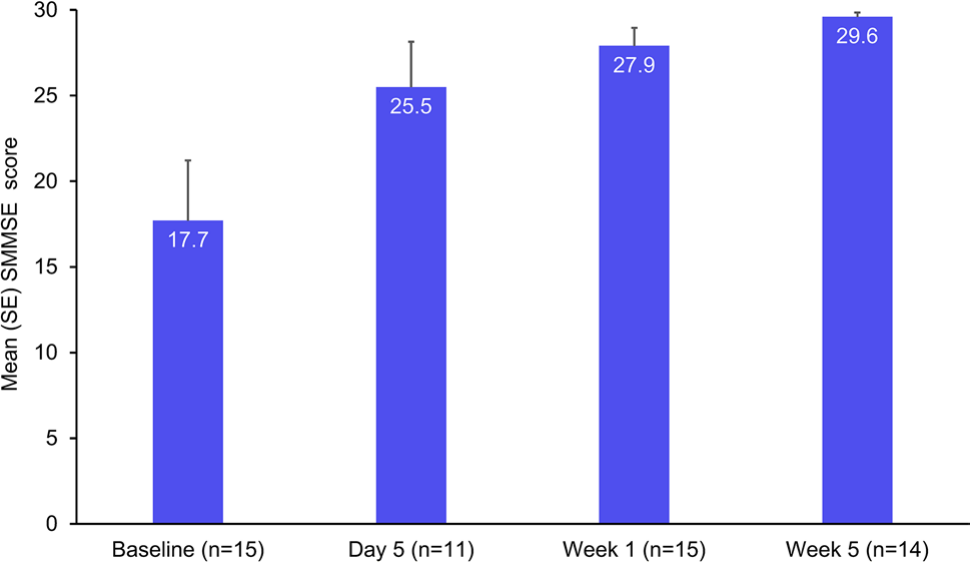
Mean (standard error) standardized Mini-Mental State Examination scores in the per-protocol population. *SE* standard error, *SMMSE* Standardized Mini-Mental State Examination

Mean (range) SMMSE scores improved steadily over the course of treatment in the PP population, from 17.7 (0–30) at baseline (i.e., moderate cognitive impairment) to 25.5 (0–30) on day 5, 27.9 (16–30) at Week 1 (i.e., mild cognitive impairment), 29.6 (27–30) at Week 5 (i.e., normal cognition), and 29.9 (29–30) at the final follow-up visit. Mean (SE) SMMSE scores are shown in Fig. 4.

#### TTP treatment course

The fresh-frozen plasma (absolute plasma volume) replaced in the PP population by TPE over 3–11 days (median 5 days) ranged between 13.4 and 50.0 L (median 24.6 L) in the overall treatment period. Median normalized plasma volume, which is TPE days multiplied by the ratio of absolute plasma volume by estimated plasma volume, was 7.5. Seven participants in the PP population had an intensive care unit (ICU) admission during the study period; durations of ICU admission ranged from 2 to 8 (median 5) days in the overall study period. All 15 participants in the PP population were admitted to hospital; the duration of hospitalization was 10–56 days (median 37) in the overall study period.

### Safety

During the overall study period, 130 TEAEs were reported in all 21 participants (100%) in the mITT population (Table 3). The maximum severity of the TEAEs was reported as “mild” in six (28.6%) participants, “moderate” in 10 (47.6%), and “severe” in five (23.8%).

**Table 3:** Safety outcomes

*n* (%)	Safety population (*N*=21)
Any TEAE	21 (100)
Treatment related	12 (57)
Most common TEAEs (reported in >15% of participants)	
Constipation	9 (43)
Insomnia	6 (29)
Allergic transfusion reaction^a^	4 (19)
Hypokalemia	4 (19)
Urticaria	4 (19)
Serious TEAE	5 (24)
Treatment related	1 (5)
TEAE leading to death	0
TEAE leading to caplacizumab interruption	5 (24)
TEAE leading to caplacizumab withdrawal	2 (10)
Bleeding event^b^	7 (33)
Epistaxis	2 (10)
GI hemorrhage	2 (10)
Pulmonary alveolar hemorrhage	1 (5)
Contusion	1 (5)
Wound hemorrhage	1 (5)
Hemorrhage subcutaneous	1 (5)
Purpura	1 (5)
Injection-site hemorrhage	1 (5)
Thromboembolic event^c^	2 (10)
Hypersensitivity reaction^c^	12 (57)
Treatment related (injection-site rash)^c^	1 (5)

*GI* gastrointestinal, *SMQ* standard Medical Dictionary for Regulatory Activities queries, *TEAE *treatment-emergent adverse event, *TTP* thrombotic thrombocytopenic purpura

^a^All four events occurred in relation to plasma exchange (fresh-frozen plasma)

^b^Based on SMQ, excluding TTP

^c^Based on SMQ

Five participants developed serious TEAEs, of which one (pulmonary alveolar hemorrhage) was considered treatment related. This serious bleeding event occurred 24 days after caplacizumab initiation and led to caplacizumab discontinuation. The participant had a complex medical history with an underlying dermatomyositis with interstitial pneumonitis. Serious TEAEs unrelated to caplacizumab were acute cholecystitis, abnormal hepatic function, hepatitis C, *Pneumocystis jirovecii* pneumonia, pneumonia aspiration, and TTP and gastrointestinal hemorrhage in one participant each. The case of TTP was considered a serious TEAE per the study protocol, and was the recurrence of iTTP in one participant already reported in the Efficacy section (above).

The most frequently reported TEAEs (in ≥ 20% of participants) by PT were constipation [*n* = 9 (42.9%)] and insomnia [*n* = 6 (28.6%); Table 3]. Twelve participants (57.1%) had a treatment-related TEAE. The most frequently reported caplacizumab-related TEAEs (in ≥ 5% of participants) were increased alanine aminotransferase, epistaxis, and gastrointestinal hemorrhage, which occurred in two participants (9.5%) each. Two treatment-emergent thromboembolic events were reported in one participant each (4.8%)—a severe cerebral infarction in one and mild deep vein thrombosis in the other—but neither was considered to be treatment related. Seven participants (33.3%) had bleeding events (Table 3), in six (28.6%) of whom the event was considered treatment related. Twelve participants (57.1%) developed hypersensitivity reactions (urticaria, allergic transfusion reaction [*n* = 4 each, 19.0%], rash [*n* = 3, 14.3%], dermatitis acneiform, infusion-related reaction, injection-site rash, and drug hypersensitivity [*n* = 1 each, 4.8%]), one of which (injection-site rash) was considered to be treatment related. No severe and/or serious hypersensitivity reactions were noted during the study. TEAEs led to treatment discontinuation in two participants (pulmonary alveolar hemorrhage in one participant and abnormal hepatic function in another).

No deaths occurred during the overall study period (treatment and follow-up), but one participant died due to a lower gastrointestinal hemorrhage that occurred 33 days after the last dose of caplacizumab and therefore after the study end (defined as 28 days of follow-up). The investigator assessed the event as unrelated to caplacizumab, but related to corticosteroids. This was the same participant who developed a pulmonary alveolar hemorrhage on Day 25. The participant had underlying dermatomyositis and interstitial pneumonitis, and had earlier (on Day 16) developed acute cholecystitis, which had required surgery. This participant remained in hospital from the time of the pulmonary hemorrhage on Day 25 until the fatal lower gastrointestinal hemorrhage on Day 61.

### Pharmacokinetics and pharmacodynamics

Plasma caplacizumab concentrations remained consistent throughout the treatment period (Supplementary Fig. S1). During the post-daily TPE period, mean plasma concentrations ranged from 611 ng/mL to 785 ng/mL.

**Fig. 5:**
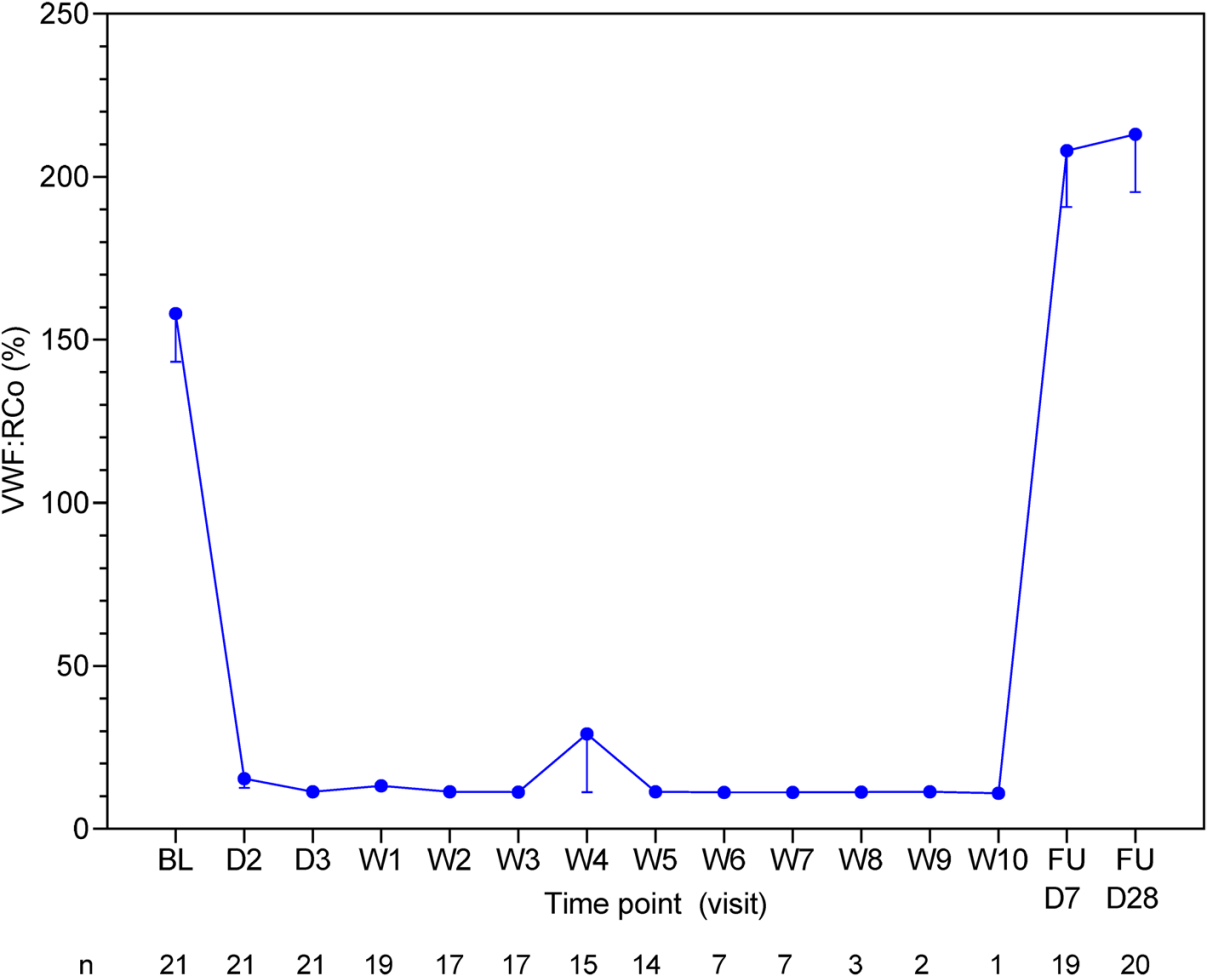
Mean plasma levels of von Willebrand factor ristocetin cofactor (VWF:RCo) in the safety population. Error bars represent standard error of the mean; D2–3 is the daily TPE period, W1–W10 is the post-daily TPE period; VWF:RCo values of < 20% represent the threshold for pharmacologic activity of caplacizumab. The mean VWF:RCo of 29.2% at Week 4 (slightly above the 20% threshold) was caused by an aberrant data point of 279.6% from one participant who stopped caplacizumab due to physician decision, *BL* baseline, *D* day, *FU* follow-up, *TPE* therapeutic plasma exchange, *W* week

Mean levels of VWF:Ag (Supplementary Fig. S2) and FVIII:C (Supplementary Fig. S3) and VWF:RCo (Fig. 5) rapidly decreased during the daily TPE period. Mean VWF:Ag levels rapidly decreased from 220% at baseline to 104% after 3 days of caplacizumab administration, thereafter remaining below baseline levels, but rapidly returning to baseline levels during the follow-up period. Mean FVIII:C levels also rapidly decreased from 205% at baseline to 158% after 3 days of caplacizumab treatment, remaining at or slightly above this level until the end of the post-daily TPE period and rapidly increasing to baseline levels during the follow-up period. With the exception of the participant who had recurrent iTTP, mean VWF:RCo decreased to below 20% (the threshold for pharmacologic activity of caplacizumab) by the first post-dose assessment time point (i.e., Day 2 of daily TPE) and remained at low levels throughout caplacizumab treatment; VWF:RCo rapidly returned to baseline levels during the follow-up period. The participant who had recurrent iTTP had elevated levels of VWF:Ag (233%), FVIII:C (220%), and VWF:RCo (100.9%) on recurrence Day 1, but these levels rapidly decreased with a second course of caplacizumab treatment.

#### ADAMTS13 activity

**Fig. 6:**
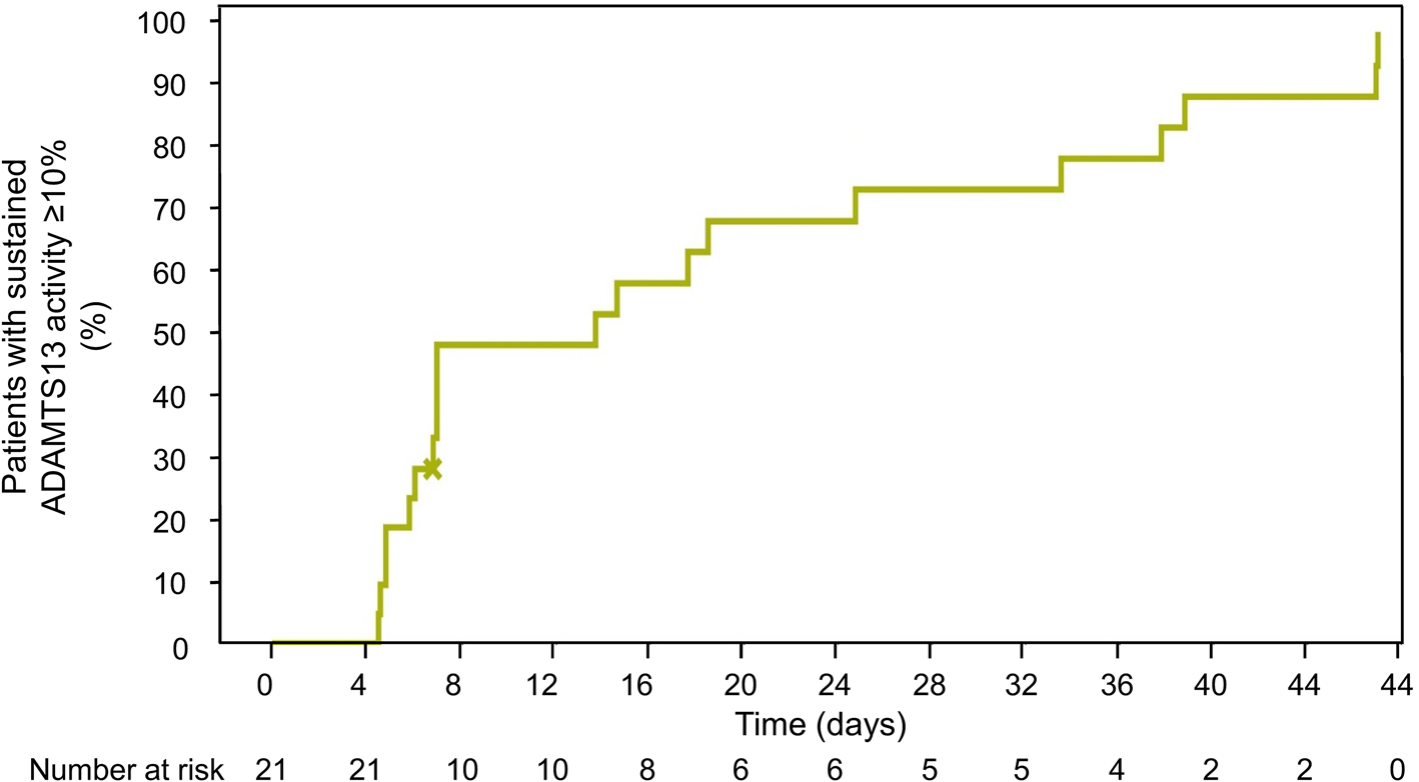
Kaplan–Meier curve of recovery of A disintegrin and metalloproteinase with a thrombospondin type 1 motif, member 13 (ADAMTS13) activity level to ≥ 10%. The x indicates a censored event

Mean ± SE ADAMTS13 activity increased steadily from 7.06 ± 6.12% at screening to 39.37 ± 7.80% at the end of Week 1, then dropped to 28.18 ± 6.76% at Week 2 (1 week after the end of TPE), and increased again to 49.5 ± 10.15% by the Week 5 visit. The participant who had iTTP recurrence had an ADAMTS13 activity level of < 1% at the time of the recurrence. ADAMTS13 activity level at the end of treatment exceeded 10% in all participants. Twenty of 21 participants achieved a sustained ADAMTS13 level of ≥ 10%, which was defined as two consecutive weekly visits at which ADAMTS13 activity was ≥ 10%, and the median time to achievement was 13.68 (95% CI 5.95–24.76) days (25% quartile 5.95 [95% CI 4.44–6.92] days; 75% quartile 33.53 [95% CI 13.69–46.90] days; Fig. 6).

### Immunogenicity

Three participants (14.3%) developed anti-caplacizumab antibodies during treatment. Neither pre-existing antibodies nor anti-drug antibodies were found to have an influence on the time to platelet count response, plasma caplacizumab concentrations, VWF:RCo, or VWF:Ag levels.

## Discussion

This study, the first to evaluate caplacizumab treatment in Japanese individuals with iTTP, indicates that adding caplacizumab to the standard protocol of TPE and immunosuppressive treatment results in a low rate of iTTP recurrence, and rapid normalization of platelet count and organ function parameters. It is notable that the participants in our study were generally older than those seen in US and European studies of individuals with iTTP (median 59 years versus 33–41 years) and included a lower proportion of females (48% vs. 77–82%) [2–4], but are consistent with previous reports on the characteristics of Japanese individuals with iTTP and severely deficient ADAMTS13 activity [15].

Only one participant in the current study had an iTTP recurrence after caplacizumab had been interrupted, so the primary endpoint occurred at a rate of 6.7%, well below the 20% rate used as a predefined criterion of success. This criterion corresponds to the number of participants fewer than three out of 15 patients (below 20%), which is set based on the expected number of participants with recurrence in our study would be 2.25 (the expected recurrence rate of 15%), assuming that capalacizumab treatment reduces relative risk (an estimated recurrence rate without capalacizumab treatment of 30%, which is based on a systematic review of past studies [16]) by 50%. The low relapse rate in our study (6.7%) is similar to the 12% relapse rate reported among caplacizumab recipients in the phase 3 HERCULES study conducted in Australia, Europe, Israel, and USA [12]. The one participant in our study who relapsed had an ADAMTS13 activity level of < 1%, which is consistent with previous data showing that relapses seem to be limited to individuals with persistently low ADAMTS13 activity levels (i.e., < 10%) [11, 12]. In our study, the underlying disease status (i.e., low ADAMTS13 activity level) led to prolonged caplacizumab treatment, as in the HERCULES study [12], which may have also contributed to the low rate of recurrence.

The favorable efficacy of caplacizumab treatment was also observed with regard to the secondary endpoints in our study. All 15 participants (100%) in the PP population and 17/21 participants (81.0%) in the mITT population experienced a rapid platelet count response, and the median times to platelet count normalization in our study (median 2.79 days in the PP population and 3.49 days in the mITT population), were similar to those observed in the TITAN and HERCULES studies [11, 12]. The short duration of TPE use in the current study (median 5 days) was also similar to those reported in the TITAN and HERCULES studies [11, 12].

Fourteen of 15 participants (93.3%) in the PP population and 18 of 21 participants (85.7%) in the mITT population had normalization of all three organ damage marker levels within a median of 2.65 days and 3.57 days, respectively. The normalization of organ damage marker levels was almost as rapid as the platelet response, suggesting that suppression of microthrombus formation during caplacizumab treatment leads to a rapid recovery from iTTP-related organ damage. In addition, rapid improvement from baseline in SMMSE scores indicated that cognitive impairment due to iTTP, present prior to the start of caplacizumab treatment, rapidly reversed after initiation of caplacizumab.

In line with data from the HERCULES and TITAN studies, none of the individuals who received caplacizumab in our study developed refractory disease. In the HERCULES study, none of the 65 caplacizumab recipients had refractory disease, but 3 of the 64 placebo recipients (4.7%) had refractory acquired TTP [12]. In addition, in an integrated analysis of data from TITAN and HERCULES, caplacizumab was associated with significantly fewer cases of refractory TTP than placebo [0 vs. 8 (7.1%); *p* < 0.01] [17].

The type and incidence of caplacizumab-related TEAEs in the current study were similar to those reported in the TITAN and HERCULES studies [11, 12] and European real-world studies [18], with no unexpected adverse events identified in this Japanese population. Bleeding events are expected during caplacizumab treatment due to the mechanism of action of the drug. Similar to previous studies, bleeding events were reported in 33% of participants in the current study, but only one serious bleeding event occurred and most bleeding events were manageable (only one participant discontinued caplacizumab treatment due to bleeding). The one participant who developed a serious bleeding event (pulmonary alveolar hemorrhage) had underlying dermatomyositis and interstitial pneumonitis. Ten percent of individuals receiving caplacizumab in the current study discontinued treatment because of TEAEs, which is similar to the rate (about 8%) in the randomized studies [11, 12].

VWF is the target molecule of caplacizumab and is also known to contribute to stabilization of FVIII. In this study, pharmacodynamic parameters, including VWF:RCo, VWF:Ag, and FVIII:C, showed a similar trend to that observed in the TITAN study [11], rapidly decreasing soon after the start of caplacizumab administration and returning to baseline levels within 1 week after the end of treatment. Levels of VWF:Ag, FVIII:C, and VWF:RCo in the TITAN study [11] were not as high as those reported in the current study. These high VWF:Ag and VWF:RCo levels may be caused by an increased rate of VWF production in the Japanese individuals with iTTP in this study, leading to higher VWF-related parameters. Older individuals tend to have higher VWF-related parameters [19]; thus, the older age of our study cohort compared with that of the TITAN study may explain the higher levels of VWF-related parameters observed in our study.

Our data support previous findings that ADAMTS13 activity is a predictor of exacerbation or recurrence. In our study, the only participant who had an iTTP recurrence had an ADAMTS13 level of < 1%. Similarly, almost all exacerbations and relapses in caplacizumab recipients in the TITAN and HERCULES studies occurred in participants with an ADMATS13 activity of < 10% at the end of treatment [11, 12]. In our study, ADAMTS13 activity levels exceeded 10% at the end of caplacizumab treatment in all participants, with rapid recovery of ADAMTS13 activity during caplacizumab. The median time from the start of caplacizumab to when the ADAMTS13 activity levels reached ≥ 10% was 13.7 days, although 75% of participants reached ≥ 10% ADAMTS13 activity after ~ 34 days. The pattern and time course of ADAMTS13 activity recovery in our study was generally consistent with previous reports in European individuals with acquired TTP [18, 20, 21], although recovery of ADAMTS13 activity in our study was faster than in a real-world German study of caplacizumab treatment, in which the median time to ADAMTS13 activity > 10% was 21 days after starting TPE (a week longer than in our study) [21]. In the German study, the slow recovery of ADAMTS13 activity was possibly because caplacizumab was often administered after TTP relapse or in cases of refractory disease [21]. In a French real-world study of caplacizumab treatment, the median time to recovery of ADAMTS13 activity to > 20% was 28 days [18].

Our study has some limitations, specifically the small sample size arising from the rarity of iTTP in clinical practice, and the lack of a control group. Given these limitations, results should be considered preliminary; however, it is important to note that they are internally consistent across endpoints, as well as being externally consistent with previously published data in mostly European individuals with TTP.

In conclusion, in Japanese individuals with iTTP, caplacizumab in combination with TPE and immunosuppressive treatment was associated with a low rate of iTTP recurrence (6.7%), which met the criterion for success, and rapid normalization of platelet counts and organ damage markers. No unexpected TEAEs were identified in the Japanese study population.

## Supplementary Information

Below is the link to the electronic supplementary material.

Supplementary file1 (DOCX 339 KB)

## Data Availability

Qualified researchers may request access to patient level data and related study documents including the clinical study report, study protocol with any amendments, blank case report form, statistical analysis plan, and dataset specifications. Patient level data will be anonymized and study documents will be redacted to protect the privacy of our trial participants. Further details on Sanofi’s data sharing criteria, eligible studies, and process for requesting access can be found at: https://www.sanofi.com/en/science-and-innovation/clinical-trials-and-results/our-data-sharing-commitments and https://www.vivli.org/.
