# Supplementary material for: The efficacy and safety of caplacizumab in Japanese patients with immune-mediated thrombotic thrombocytopenic purpura: an open-label phase 2/3 study
Source: Int J Hematol. 2022 Nov 24;117(3):366–77. doi: 10.1007/s12185-022-03495-6 (PMC9970947; doi:10.1007/s12185-022-03495-6)

**The efficacy and safety of caplacizumab in Japanese individuals with immune-mediated thrombotic thrombocytopenic purpura: an open-label phase 2/3 study**

**Supplementary Material**

**Supplementary methods**

**Treatment**

Corticosteroids: methylprednisolone intravenous (IV) pulse therapy was initiated at 1 g/day for 3 days, or a prednisolone regimen of IV or oral 1 mg/kg/day was administered during the daily therapeutic plasma exchange (TPE) period and continued for the first week after the end of daily TPE. Corticosteroids were subsequently tapered at the discretion of the investigator, with the aim of discontinuation by Day 30 after the cessation of daily TPE.

**Assessments**

Organ damage markers were lactate dehydrogenase, cardiac troponin I, and serum creatinine. Pharmacodynamic parameters included von Willebrand factor (VWF) ristocetin cofactor (VWF:RCo), VWF antigen (VWF:Ag), and coagulation factor VIII clotting activity (FVIII:C). VWF:RCo measurements were used to assess VWF (target factor) activity, and VWF:Ag was used as a target pharmacokinetic marker. Anti-drug antibody serum levels were evaluated as described previously [1], with some modifications. Blood samples were taken for pharmacokinetic analysis on Days, 1, 2, and 3, then weekly until the end of the study. Anti-drug antibodies were measured at baseline, Week 1, Week 5 (or end of study), and at the first and last follow-up visits.

**Outcomes**

The severity of treatment-emergent adverse events (TEAEs) was classified as "mild" (i.e., an event easily tolerated by the participant, causing minimal discomfort and not interfering with everyday activities), "moderate" (i.e., an event that causes sufficient discomfort to interfere with normal everyday activities), or "severe" (i.e., an event that prevents normal everyday activities). Adverse events (AEs) assessed as “severe” were distinct from serious AEs (SAEs). Severe is a category utilized for rating the intensity of an event; both AEs and SAEs can be assessed as severe. A TEAE was defined as “serious” when it met at least one of the predefined outcomes describing the definition of a SAE, not when it was rated as severe.

1. Peyvandi F, Scully M, Kremer Hovinga JA, Cataland S, Knobl P, Wu H, et al. Caplacizumab for acquired thrombotic thrombocytopenic purpura. N Engl J Med. 2016;374:511-22.

**Supplementary Figures**

**Supplementary Fig S1** Mean plasma concentrations of caplacizumab in the modified intent-to-treat population. Error bars represent standard error of the mean. D2–3 was the daily therapeutic plasma exchange (TPE) period, W1–W10 was the post-daily TPE period.

*D* day; *W* week.


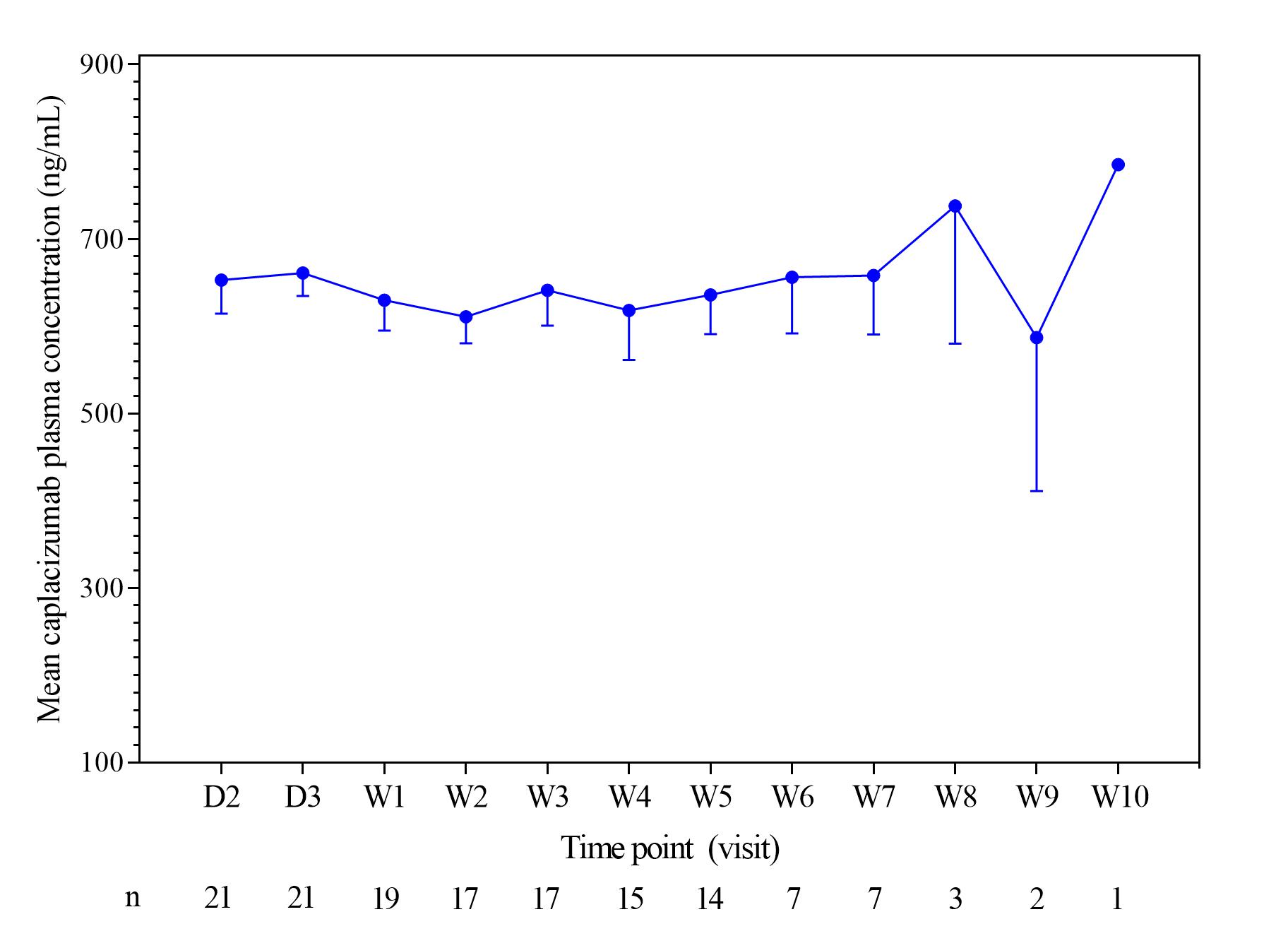


**Supplementary Fig S2** Mean plasma levels of von Willebrand factor antigen (VWF:Ag) over time in the safety population. Error bars represent the standard error of the mean. D2–3 was the daily therapeutic plasma exchange (TPE) period, W1–W10 was the post-daily TPE period.

*BL* baseline; *D* day; *FU* follow-up; *W* week.


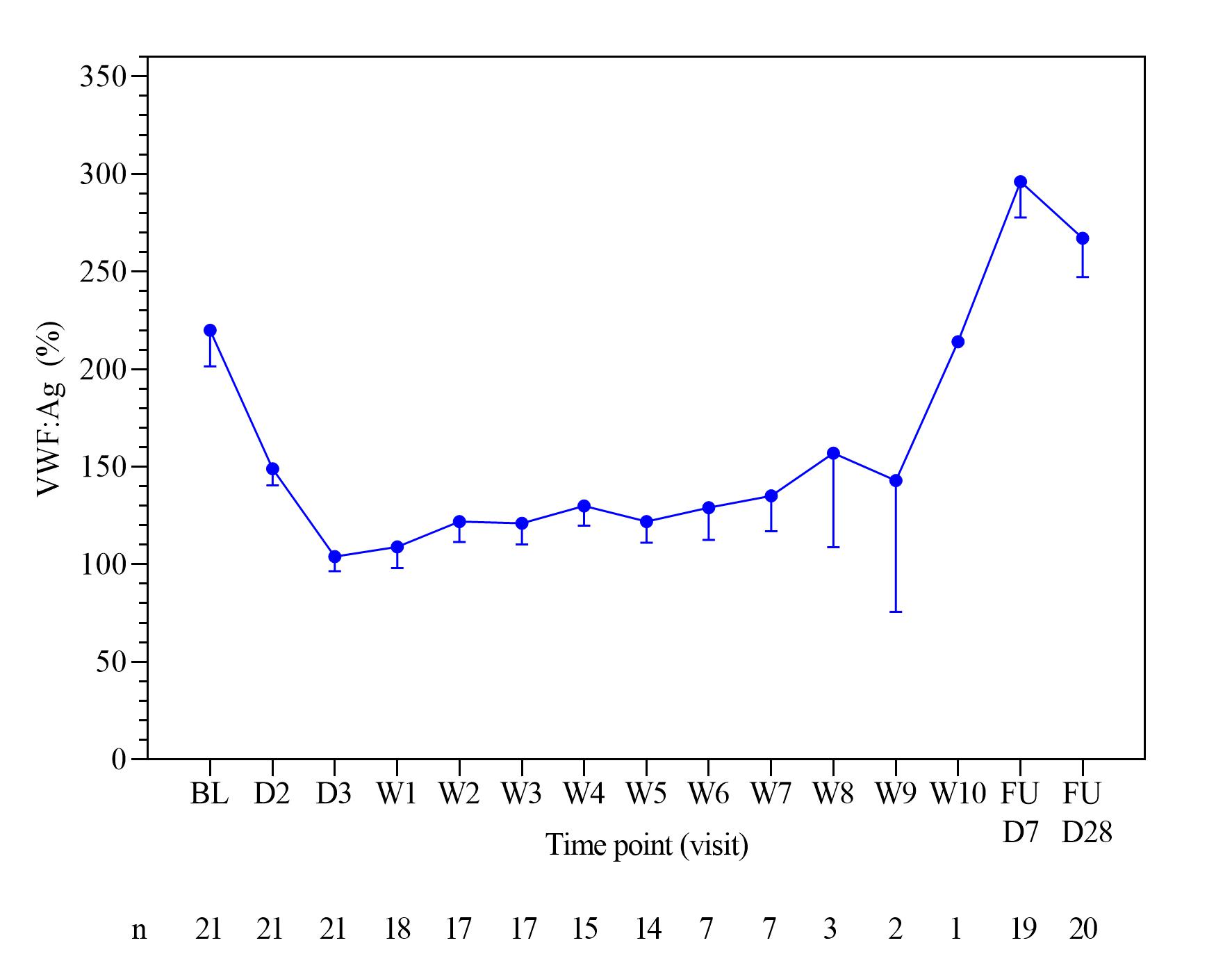


**Supplementary Fig S3** Mean plasma levels of factor VIII clotting activity (FVIII:C) over time in the safety population. Error bars represent the standard error of the mean. D2–3 was the daily therapeutic plasma exchange (TPE) period, W1–W10 was the post-daily TPE period.

*BL* baseline; *D* day; *FU* follow-up; *W* week.


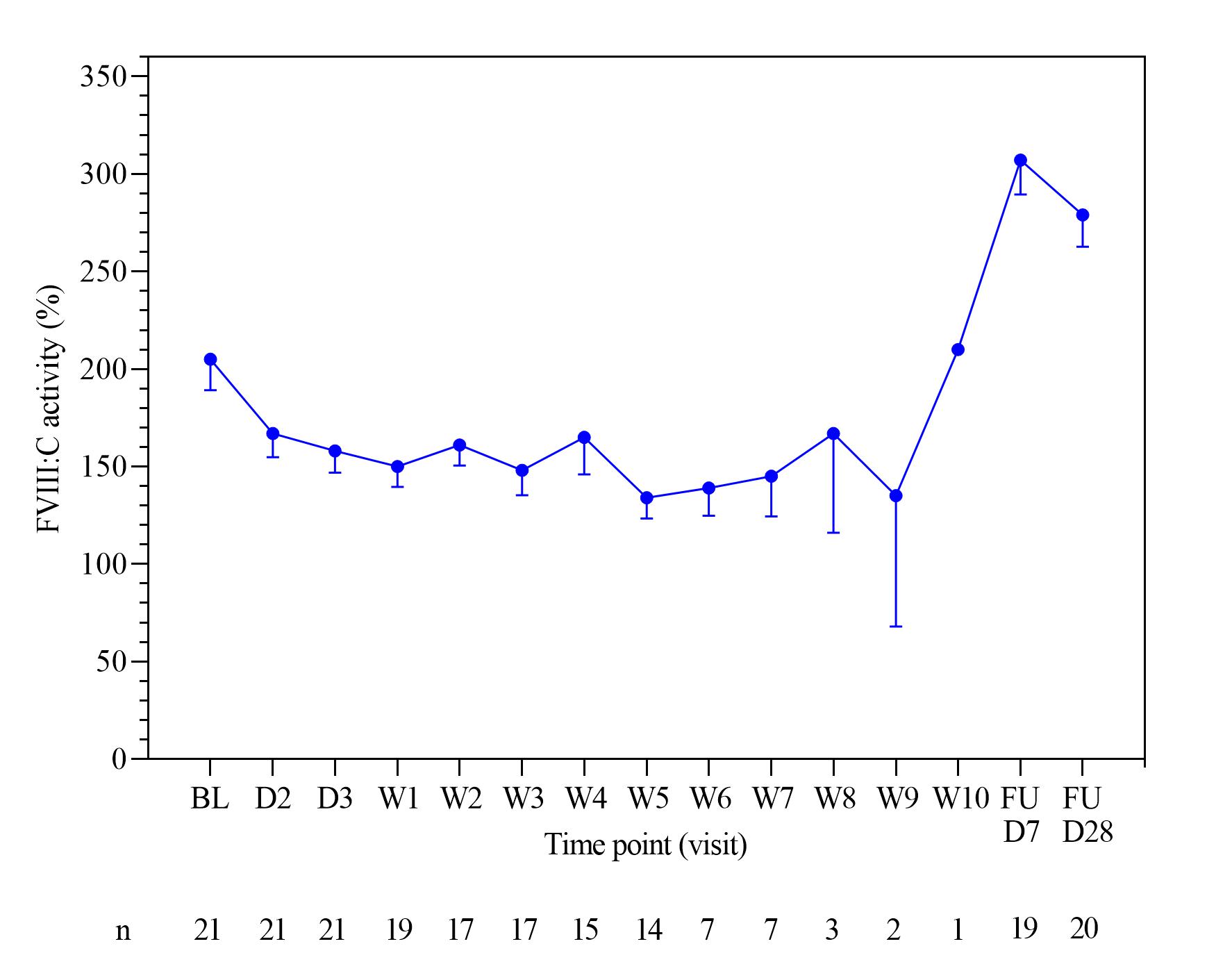

Supplement: Supplementary file 1 — Supplementary file1 (DOCX 339 KB) [file 12185_2022_3495_MOESM1_ESM.docx]
